# Molecular diagnosis of African tick bite fever using eschar swabs in a traveller returning from Tanzania

**DOI:** 10.1007/s00508-016-1047-0

**Published:** 2016-08-03

**Authors:** Nicole Harrison, Heinz Burgmann, Christina Forstner, Michael Ramharter, Marton Széll, Anna‑Margarita Schötta, Gerold Stanek, Mateusz Markowicz

**Affiliations:** 1 Department of Medicine I, Division for Infectious Diseases and Tropical Medicine, Medical University of Vienna, Vienna, Austria; 2 Center of Infectious Diseases and Infection Control, Jena University Hospital, Jena, Germany; 3 Institute for Tropical Medicine, University of Tübingen, Tübingen, Germany; 4 Department of Medicine II, Division for Emergency Medicine, SMZ Ost – Donauspital, Vienna, Austria; 5 Institute for Hygiene and Applied Immunology, Medical University of Vienna, Kinderspitalgasse 15, 1090 Vienna, Austria

**Keywords:** African tick bite fever, Tanzania, *Rickettsia africae*, Rickettsiosis, Eschar

## Abstract

African tick bite fever is an emerging infectious disease among travellers caused by the pathogen *Rickettsia africae*. Most travel-associated cases have been reported from countries in southern Africa. So far it has rarely been reported among travellers to eastern Africa and our patient is one of the first described cases imported from Tanzania. A woman presented with fever, chills, headache, myalgia and a rickettsial eschar on her ankle after returning from Tanzania. The diagnosis of African tick bite fever is often based on clinical grounds due to a lack of reliable diagnostic tests at commencement of symptoms. In this patient direct molecular detection of *R. africae* was performed by PCR from a sample obtained non-invasively with a swab from the rickettsial eschar. A positive PCR result was achieved although the patient had already started antibiotic treatment with doxycycline. In conclusion, this non-invasive method enables early diagnosis of African tick bite fever by direct molecular detection of *R. africae* and might improve the management of undifferentiated fever in travellers from Africa.

## Introduction

African tick bite fever (ATBF) is an emerging infectious disease among travellers. In febrile travellers returning from sub-Saharan Africa rickettsial infections were the second most common etiology after malaria [1]. *Rickettsia africae* was the most frequently detected pathogen in tick-bite related diseases from Africa [2]. *R. africae* was first identified as a new human pathogen in 1992 [3] and since then has been isolated from ticks of different *Amblyomma *species from many regions across Africa [4, 5]. Most travel-associated cases of ATBF have been reported from South Africa and adjoining countries especially among game hunters [6]. So far ATBF has rarely been reported among travellers to East Africa and our patient is one of the first described cases imported from Tanzania. This fact could be explained by various vectors of *R. africae*, which might result in different risks of transmission. In eastern African countries *R. africae* is commonly found in ticks of the species *Amblyomma variegatum *[5], whereas in South Africa *Amblyomma hebraeum* is known to transmit the pathogen. Furthermore, different variants of *R. africae* detected in eastern Africa might be less pathogenic for humans [5, 6].

We describe a case of ATBF with direct molecular detection of *R. africae* by PCR from a sample obtained non-invasively from the rickettsial eschar. Considering that cases of ATBF imported from Tanzania are rarely reported, we aimed to sequence the detected *R. africae* to determine the exact genotype of this pathogen.

## Case report

**Fig. 1:**
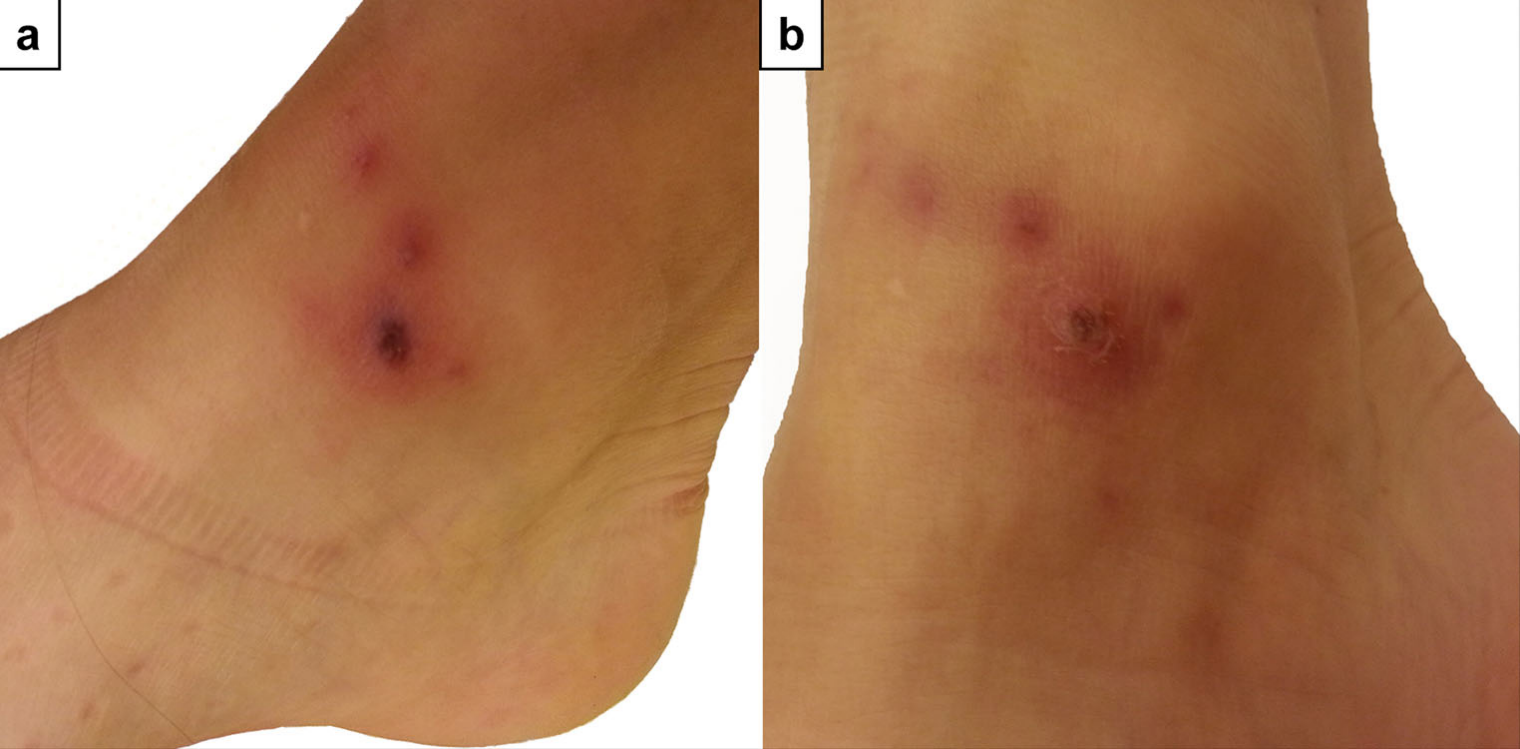
The rickettsial eschar on the right ankle of the patient during the first visit before treatment was initiated (**a**) and at the follow-up 1 week later after treatment with doxycycline (**b**)

A 30-year-old woman presented with fever, chills, headache and myalgia at the outpatient ward for tropical medicine at the University Hospital of Vienna. A skin lesion on the right ankle and a swollen inguinal lymph node were noticed on physical examination. The patient had recently returned from working on a developmental aid project in the southwest of Tanzania. She had spent 2 weeks living in a small rural village and hiking through the adjoining national parks. On a trip through the Kipengere Mpanga Game Reserve she had noticed a tick bite on the right ankle and 1 week later a reddish skin lesion developed at the site of the bite. The next day the patient experienced high fever (39 °C), chills, headache, generalized myalgia and pain in the right lower limb. On the second day of fever the patient visited our outpatient ward. The laboratory analysis showed a moderate leukopenia (3.5 × 10^3^/µl) with an otherwise unremarkable blood count and without elevation of inflammation parameters. A rapid diagnostic test and a thick blood film for malaria were negative. The skin lesion on the right ankle was typical for a rickettsial eschar (Fig. 1) and the diagnosis of ATBF was established on clinical grounds. The patient received 200 mg doxycycline daily for 9 days and the fever resolved after 2 days of treatment. Other symptoms, such as headache, muscle pain and inflammation at the site of the tick bite improved after 1 week of administering doxycycline.

**Fig. 2:**
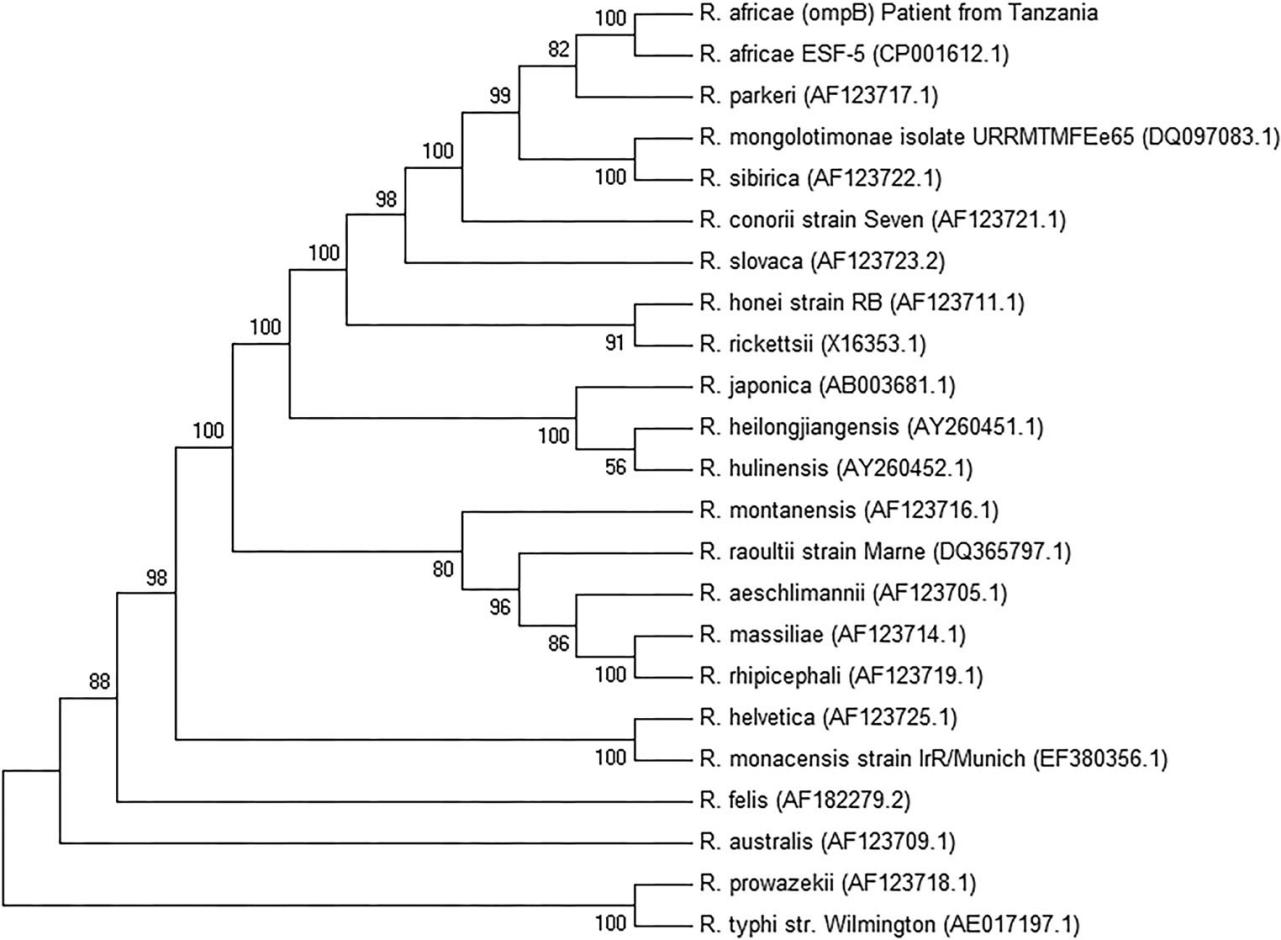
Phylogenetic tree based on partial *ompB* nucleotide sequences of the genus *Rickettsia*. The tree was constructed by using the maximum likelihood method with the use of the Tamura-3-parameter model and gamma distribution. The bootstrap consensus tree was inferred from 1000 replicates. A total of 23 nucleotide sequences were involved in the analysis. Accession numbers of sequences obtained from GenBank are shown in parenthesis. The final dataset included a total of 4155 positions. Evolutionary analysis was conducted in MEGA (Molecular Evolutionary Genetics Analysis) version 7.0

For further diagnostic work-up serological testing for IgG and IgM against *R. conorii* was performed at initial presentation. Both the immunofluorescence assay (Focus Diagnostics, Cypress, CA) and the Weil Felix agglutination assay (DiaMondial, Sees, France) gave negative results. These serological tests are designed to detect *R. conorii* but may also give positive results for *R. africae* due to cross-reactivity. Furthermore, PCR performed on DNA extracted from an EDTA blood sample yielded a negative result for *Rickettsia *spp. At a follow-up visit 5 days later, the crust of the eschar was removed and two samples from the base of the lesion were taken with a dry swab to perform PCR for *Rickettsia spp*. At that time treatment with doxycycline had already been administered for 5 days. A serological follow-up was performed on day 17 after the tick bite and seroconversion of IgG was observed in the immunofluorescence assay (titre 1:512). For PCR testing of the eschar samples, DNA was extracted from each sample using the PeqGOLD Tissue DNA Mini Kit (Peqlab, Erlangen, Germany) and carried out according to the manufacturer’s protocol. For the detection of *Rickettsia *spp. samples were subjected to a real time PCR targeting the *gltA* gene [7]. *Rickettsia* DNA could be detected in one of the two swabs. To further identify the species present in the sample, additional PCR targeting the 16S rRNA gene [8], 23S-5S intergenic spacer [9], *gltA* gene [10] and *ompB* gene [11] were performed as previously described. Primers and probes used are displayed in Table 1. Amplicons were purified using QIAquick PCR purification kit and Qiagen gel extraction kit (Qiagen, Hilden, Germany) if multiple bands were observed. Purified products were sent to MWG (Eurofins, Ebersberg, Germany) for bidirectional sequencing. Consensus sequences were created and *ompB* fragments were assembled by using CLC Main Workbench (version 7.6). A BLAST search (http://blast.ncbi.nlm.nih.gov/Blast.cgi) yielded a 100 % identity to *R. africae* strain ESF-5. The sequences obtained in this study were submitted to GenBank (accession numbers: KU721068, KU721069, KU721070, KU721071). A maximum likelihood-based phylogenetic tree (Fig. 2) for the obtained *ompB* sequence was constructed by using the analysis software package MEGA7 (version 7.0.14).

**Table 1:** Primer and probe sequences used to identify *Rickettsia africae*

Oligonucleotide name	Target gene	Oligonucleotide sequence (5’ → 3’)	Reference
*Real time PCR*			
RickF	gltA gene	GGT ATA CCG TCG CAA ATG TTC AC	[7]
RickR	gltA gene	GGG TCT TCG TGCATT TCT TTC C	[7]
RickTM	gltA gene	TGT GCC ATC CAG CCT ACG GTT CTT G^a^	[7]
*Primers used for sequencing*			
Rick-F1	16S rRNA gene	GAA CGC TAT CGG TAT GCT TAA CAC A	[8]
Rick-R2	16S rRNA gene	CAT CAC TCA CTC GGT ATT GCT GGA	[8]
RCK/23-5-F	23S-5S IGS	GAT AGG TCR GRT GTG GAA GCA C	[9]
RCK/23-5-R	23S-5S IGS	TCG GGA YGG GAT CGT GTG TTT C	[9]
CS-78	gltA gene	GCA AGT ATC GGT GAG GAT GTA AT	[10]
CS-323	gltA gene	GCT TCC TTA AAA TTC AAT AAA TCA GGA T	[10]
M59	ompB (I)	CCGCAGGGTTGGTAACTGC	[11]
120-807 R	ompB (I)	CCTTTTAGATTACCGCCTAA	[11]
120-607 F	ompB (II)	AATATCGGTGACGGTCAAGG	[11]
120-1497	ompB (II)	CCTATATCGCCGGTAATT	[11]
120-1378	ompB (III)	TAAACTTGCTGACGGTACAG	[11]
120-2399	ompB (III)	CTTGTTTGTTTAATGTTACGGT	[11]
120-2113	ompB (IV)	CGATGCTAACGTAGGTTCTT	[11]
120-2988	ompB (IV)	CCGGCTATACCGCCTGTAGT	[11]
120-2788	ompB (V)	AAACAATAATCAAGGTACTGT	[11]
120-3599	ompB (V)	TACTTCCGGTTACAGCAAAGT	[11]
120-3462	ompB (VI)	CCACAGGAACTACAACCATT	[11]
120-4346	ompB (VI)	CGAAGAAGTAACGCTGACTT	[11]

^a^Real time PCR probe was labeled with 5’FAM and 3’TAMRA

## Discussion

The diagnosis of ATBF is often based on the clinical presentation, especially on the presence of fever and one or multiple eschars. A confirmatory laboratory method is necessary to differentiate ATBF from other febrile diseases. For serological testing the immunofluorescence test is the preferred method but seroconversion usually occurs about 2–3 weeks after the tick bite; therefore, serological testing is often negative at the onset of symptoms as shown in our patient. The use of PCR on skin biopsy samples from the eschar has been proposed as a method with high sensitivity [2]; however, obtaining a skin biopsy may not be practical in certain settings. As an alternative the use of a non-invasive eschar swab has proven to be effective in several case series [12–14]. The eschar crust should be removed and a dry swab circulated on the base of the eschar to yield a sufficient sample. This method also showed high acceptance among physicians and patients [15]. Both the sampling and the transport (optimal temperature 4 °C) are easy to perform for research in field conditions and therefore a good alternative to the more laborious skin biopsy [13]. In the described case one of the two eschar swabs contained sufficient material for a positive PCR result although our patient had already received treatment with doxycycline for 5 days.

In conclusion, this case confirms the usefulness of eschar swabs to detect the agent of ATBF by PCR even after starting antibiotic treatment. Finally, ATBF must be considered in febrile travellers returning from East Africa, although imported infections have rarely been reported so far from this part of Africa.

## References

[1] Freedman DO, Weld LH, Kozarsky PE, Fisk T, Robins R, von Sonnenburg F (2006). Spectrum of disease and relation to place of exposure among ill returned travelers. N. Engl. J. Med..

[2] Raoult D, Fournier PE, Fenollar F, Jensenius M, Prioe T, de Pina JJ (2001). Rickettsia africae, a tick-borne pathogen in travelers to sub-Saharan Africa. N. Engl. J. Med..

[3] Kelly PJ, Beati L, Matthewman LA, Mason PR, Dasch GA, Raoult D (1994). A new pathogenic spotted fever group rickettsia from Africa. J Trop Med Hyg.

[4] Lorusso V, Gruszka KA, Majekodunmi A, Igweh A, Welburn SC, Picozzi K (2013). Rickettsia africae in Amblyomma variegatum ticks, Uganda and Nigeria. Emerging Infect. Dis..

[5] Maina AN, Jiang J, Omulo SA, Cutler SJ, Ade F, Ogola E (2014). High prevalence of Rickettsia africae variants in Amblyomma variegatum ticks from domestic mammals in rural western Kenya: implications for human health. Vector Borne Zoonotic Dis.

[6] Jensenius M, Fournier PE, Vene S, Hoel T, Hasle G, Henriksen AZ (2003). African tick bite fever in travelers to rural sub-Equatorial Africa. Clin. Infect. Dis..

[7] Leschnik MW, Khanakah G, Duscher G, Wille-Piazzai W, Horweg C, Joachim A (2012). Species, developmental stage and infection with microbial pathogens of engorged ticks removed from dogs and questing ticks. Med. Vet. Entomol..

[8] Nijhof AM, Bodaan C, Postigo M, Nieuwenhuijs H, Opsteegh M, Franssen L (2007). Ticks and associated pathogens collected from domestic animals in the Netherlands. Vector Borne Zoonotic Dis.

[9] Jado I, Escudero R, Gil H, Jimenez-Alonso MI, Sousa R, Garcia-Perez AL (2006). Molecular method for identification of Rickettsia species in clinical and environmental samples. J. Clin. Microbiol..

[10] Labruna MB, Whitworth T, Horta MC, Bouyer DH, McBride JW, Pinter A (2004). Rickettsia species infecting Amblyomma cooperi ticks from an area in the state of Sao Paulo, Brazil, where Brazilian spotted fever is endemic. J. Clin. Microbiol..

[11] Roux V, Raoult D (2000). Phylogenetic analysis of members of the genus Rickettsia using the gene encoding the outer-membrane protein rOmpB (ompB). Int J Syst Evol Microbiol.

[12] Socolovschi C, Renvoise A, Brouqui P, Parola P, Raoult D (2012). The use of eschar swabs for the diagnosis of African tick-bite fever. Ticks Tick Borne Dis.

[13] Bechah Y, Socolovschi C, Raoult D (2011). Identification of rickettsial infections by using cutaneous swab specimens and PCR. Emerging Infect. Dis..

[14] Wang JM, Hudson BJ, Watts MR, Karagiannis T, Fisher NJ, Anderson C (2009). Diagnosis of Queensland tick typhus and African tick bite fever by PCR of lesion swabs. Emerging Infect. Dis..

[15] Mouffok N, Socolovschi C, Benabdellah A, Renvoise A, Parola P, Raoult D (2011). Diagnosis of rickettsioses from eschar swab samples, Algeria. Emerging Infect. Dis..

